# Comparison of biomechanical parameters in lower limb joints of stroke patients according to conventional evaluation scores during level walking

**DOI:** 10.3389/fbioe.2024.1320337

**Published:** 2024-02-26

**Authors:** HyeongMin Jeon, Eun-Hye Chung, Se-Young Bak, Heegoo Kim, Seyoung Shin, Hongseok Baek, MinYoung Kim

**Affiliations:** ^1^ Digital Therapeutics Research Team, CHA Bundang Medical Center, CHA Future Medicine Research Institute, CHA University School of Medicine, Seongnam, Republic of Korea; ^2^ Department of Rehabilitation Medicine, CHA Bundang Medical Center, CHA University School of Medicine, Seongnam, Republic of Korea

**Keywords:** stroke, gait, kinetics, kinematics, functional ambulation category, motion analysis, affected side, unaffected side

## Abstract

**Objective:** Patients with chronic stroke capable of independent gait were classified into functional ambulation category (FAC) 4 or 5, and the kinetic and kinematic data on their lower limb joints on the affected and unaffected sides were compared with that of healthy individuals. Finally, the qualitative changes in the gait of patients with stroke were investigated based on the differences in FAC scores.

**Methods:** Twelve healthy participants and 19 patients with stroke capable of independent gait were included. The three-dimensional (3D) motion analysis and conventional assessment were conducted for all patients with stroke.

**Results:** The FAC 5 group exhibited a larger range of motion (ROM) than the FAC 4 group in knee and hip joints on the affected side and only in the hip on the unaffected side. In the FAC 5 group, ROM differences in the healthy group on either the affected or unaffected side were absent. The peak of the hip flexion moment on the affected side in both the FAC 4 and 5 groups was smaller than that in the healthy group and in the FAC 4 group on the unaffected side. The absorption power minimum on the affected side was smaller only in the FAC 4 group than that in the healthy group and was larger in the FAC 5 group than that in the FAC 4 group. On the unaffected side, the absorption power minimum was smaller only in the FAC 4 group than that in the healthy group.

**Conclusion:** Functional differences in gait were found in patients classified based on conventional evaluation capable of independent gait after post-stroke rehabilitation. Patients may not exhibit complete recovery in the kinetic indices even if they are judged to be normal in the conventional evaluation, and the kinematic gait indices indicate recovery. Evaluating kinetic indices in addition to kinematic indices is necessary, and joint power may be an especially useful index.

## 1 Introduction

More than 10 million cases of stroke are reported per year worldwide, and the disease is especially frequent in elderly patients (Seshadri et al., 2006; Sousa et al., 2009; Feigin et al., 2015). Stroke is one of the major causes of death and long-term disability (Sousa et al., 2009; Feigin et al., 2014; Saini et al., 2021).

Most patients with stroke suffer from sequelae such as motor control challenges or restricted mobility due to hemiplegia, leading to impairments in daily living (Gresham et al., 1995; Woolley, 2001; Kim et al., 2016; Benjamin et al., 2019). Various types of rehabilitation therapy are used to restore daily living functions. In particular, the recovery of gait function is a major goal in rehabilitation to help patients with stroke return to daily living (Olney and Richards, 1996; Wonsetler and Bowden, 2017; Bigoni et al., 2021). Over 70% of patients with stroke cannot independently walk at first after their injury; however, after 6 months of appropriate rehabilitation therapy, the percentage of patients capable of independent gait can increase up to 85% (Von Schroeder et al., 1995; Woolley, 2001).

As such, numerous studies using conventional assessment methods, such as the Timed Up and Go (TUG) (Podsiadlo and Richardson, 1991), 6-min walk (Enright, 2003), functional ambulation category (FAC) (Corrigan et al., 1997), functional independence measure (FIM) (Mehrholz et al., 2007), and 10-meter walk test (10MWT) (Graham et al., 2008), have been conducted to identify pathologic gait patterns in patients with stroke, establish rehabilitation strategies, and investigate how different interventions affect gait function recovery. Patients who had recovered functional gait after stroke exhibited an increased risk of falls compared with healthy individuals. Changes differ even between recovered patients, and decreased gait speed and increased stance phase are generally more severe on the affected than unaffected sides. However, these findings do not provide sufficient qualitative evidence regarding gait mechanisms and symmetry (Shin et al., 2020). Consequently, several studies have been conducted using three-dimensional (3D) motion capture, oxygen consumption measures, or electromyography (Olney and Richards, 1996; Wonsetler and Bowden, 2017; Fotiadou et al., 2019; Mentiplay et al., 2019; Bigoni et al., 2021). In particular, when used with a force plate, 3D motion capture provides biomechanical data, allowing for the quantitative and accurate measurement of the gait function of patients (Latorre et al., 2018; Ferraris et al., 2021).

Gait, one of the most frequently performed and essential human movements, is achieved through a complex coordination between different muscles, tendons, and ligaments (Ferris et al., 1998; Kuo, 2007). Previous studies have attempted to understand the mechanism of gait, even in healthy individuals. In the bi-supported segment of level walking, depending on the energy changes in the whole body, the leading leg strikes the ground and dissipates mechanical energy through absorption power, whereas the trailing leg moves the center of mass (COM) up and down through energy generation power (Donelan et al., 2002; Franz et al., 2012). During this process, joints affect each other, and the mechanical energy generation and mechanical energy absorbed by the net moment of each joint is transferred between body segments to affect each joint (Caldwell and Forrester, 1992; Siegel et al., 2004; Umberger et al., 2013). For example, during gait, the plantar flexor moment at the ankle transfers energy between the thigh and trunk, allowing forward movement (Siegel et al., 2004). Therefore, understanding the kinetic index of each joint during gait is the first step in understanding gait pathology (Umberger et al., 2013).

The 3D motion capture data can provide a wealth of data to understand gait pathology, thereby providing novel insights into patients’ conditions, which in turn enables personalized and effective rehabilitation. Previous stroke studies using 3D motion capture have mostly focused on kinematic data, reporting pathological characteristics such as decreased flexion angle at the hip joint, hyperextension at the knee joint, and reduced dorsiflexion angle at the ankle joint (Olney and Richards, 1996; Balaban and Tok, 2014; Wonsetler and Bowden, 2017; Shin et al., 2020; Karunakaran et al., 2022).

Another biomechanical data type, kinetic data, which include the moment and power generated at the joints, can help determine the muscle strength and movement patterns at the major joints in patients with stroke and provide insights into the causes of gait disorders (Olney et al., 1991; Sheffler and Chae, 2015). However, studies that have used kinetic indices to investigate the pathological characteristics in patients with stroke are limited, and most studies have aimed to explain the effects of the interventions. Moreover, the inclusion criteria, such as “capable of independent gait,” used for selecting participants are unclear (Olney et al., 1991; Olney and Richards, 1996; Balaban and Tok, 2014; Wonsetler and Bowden, 2017). In particular, studies categorizing the gait function of patients with stroke based on conventional assessment methods and subsequently analyzing the kinetic indices in these patients are limited.

Therefore, this study classified patients with chronic stroke capable of independent gait into FAC 4 or 5. Next, we used the kinetic and kinematic data on the lower limb joints of patients with stroke on their affected and unaffected sides and compared them with those of healthy individuals. Finally, the qualitative changes in the gait of patients with stroke were investigated based on the differences in the conventional clinical assessment scores to provide insights into effective rehabilitation strategies.

## 2 Materials and methods

### 2.1 Participants

Twelve healthy male participants without cerebral, neural, or musculoskeletal disease participated in this study. The patients with stroke consisted of 19 men with chronic stroke, who were capable of independent gait (FAC ≥4) and for whom at least 6 months had passed since the initial stroke onset. Tables 1, 2 present the basic information and conventional assessment outcomes of the participants. Only patients whose affected and unaffected sides were distinguishable were included based on a clinician’s diagnosis. Patients with a history of musculoskeletal surgery that could affect gait were excluded. Individuals with neurological conditions other than stroke were also excluded and confirmed by a clinician to be typical stroke patients via MRI examination. The electronic medical record of CHA Bundang Medical Center was used to confirm the patients’ history. Furthermore, pre-interviews with patients were conducted to check their will on participating in the study and their physical condition, and their medical history was checked. All participants (Korean) provided written informed consent, and this study was approved by the Institutional Review Board of CHA Bundang Medical Center (Clinical trial No. NCT05908994; IRB No. 2021-05-026).

**TABLE 1:** Characteristics of the subjects.

	FAC 4 (*n* = 8)	FAC 5 (*n* = 11)	Normal (*n* = 12)
Age (years)	64.13 ± 6.53	57.00 ± 14.00	41.92 ± 13.79
Height (cm)	165.90 ± 4.56	165.20 ± 5.41	172.01 ± 5.47
Weight (kg)	64.59 ± 8.36	68.14 ± 9.51	70.38 ± 8.63
BMI (kg/m^2^)	23.51 ± 3.30	24.91 ± 2.83	23.77 ± 2.56
L foot length (cm)	24.13 ± 8.57	23.95 ± 0.92	24.85 ± 1.55
R foot length (cm)	24.13 ± 8.58	24.03 ± 0.96	24.86 ± 1.56

Continuous values are presented as the mean ± standard deviation.

**TABLE 2:** Conventional assessment outcomes and the spatio–temporal index.

Index		Subject group			p		
		FAC 4 (4)	FAC 5 (5)	Normal (Nor)	4 vs. 5	4 vs. Nor	5 vs. Nor
Spatio–temporal	Walking speed (m/s)	0.70 ± 0.07	0.91 ± 0.09	1.04 ± 0.15	.015*	.000***	.102
	Stride length(m)	0.77 ± 0.12	1.00 ± 0.15	1.12 ± 0.13	.036*	.000***	.223
Motor Assessment Scale (MAS)		37.25 ± 7.98	47.55 ± 1.51		.000***		
Timed Up and Go (TUG)		25.33 ± 12.34	11.01 ± 2.91		.002**		
Motricity Index (MI)	Unaffected leg	100.00 ± 0.00	100.00 ± 0.00		1		
	Affected leg	64.75 ± 10.90	87.55 ± 13.75		.000***		
Passive rande of motion (PROM) (deg)	Unaffected						
	Hip flexion	100.00 ± 0.00	100.00 ± 0.00		1		
	Hip extension	10.00 ± 0.00	10.00 ± 0.00		1		
	Knee flexion	110.00 ± 0.00	110.00 ± 0.00		1		
	Knee extension	0.00 ± 0.00	0.00 ± 0.00		1		
	Ankle dorsiflexion	10.00 ± 0.00	10.00 ± 0.00		1		
	Ankle plantarflexion	20.00 ± 0.00	20.00 ± 0.00		1		
	Affected						
	Hip flexion	98.75 ± 3.54	100.00 ± 0.00		.252		
	Hip extension	10.00 ± 0.00	10.00 ± 0.00		.409		
	Knee flexion	110.00 ± 0.00	110.00 ± 0.00		1		
	Knee extension	0.00 ± 0.00	0.00 ± 0.00		1		
	Ankle dorsiflexion	9.38 ± 1.77	10.00 ± 0.00		.252		
	Ankle plantarflexion	20.00 ± 0.00	20.00 ± 0.00		1		

Continuous values are presented as the mean ± standard deviation. *p < 0.05, **p < 0.01, and ***p < 0.001.

### 2.2 Clinical evaluation

The evaluation was performed by physical therapists or researchers who have been trained routinely. Criteria and methods were followed as per the CHA Bundang Medical Center clinical system.

#### 2.2.1 Functional ambulation category

The FAC was used to assess clinical gait levels. In the FAC, levels 1–3 indicated a state of dependent capability in walking, while levels 4 and 5 indicated a state of independent capability in walking. Furthermore, FAC 4 was defined as a case demanding supervision to navigate environments such as stairs and inclines despite the independent capability of ambulation on the surface level. FAC 5 was defined as a case of having independent capability of ambulation in whole environments (Mehrholz et al., 2007).

#### 2.2.2 Motor assessment scale

The motor assessment scale (MAS), a reliable and validated test for patients with stroke, was used to assess the functional motor on the affected side (Poole and Whitney, 1988). The test consists of eight items (Gor-García-Fogeda et al., 2014): supine to side-lying, lying to sitting on the bed, balance in the sitting position, sitting to standing, the function of the upper extremity, hand movements, advanced hand activity, and walking. Each item was scored with a point of 0–6 (Carr et al., 1985), where higher scores indicate better motor function.

#### 2.2.3 Motricity Index

The Motricity Index (MI) is used to measure muscle force to predict mobility outcomes post-stroke (Fayazi et al., 2012). In this study, scores of lower extremities were measured and analyzed. The movements considered for the evaluation of lower extremities were hip flexion, knee extension, and ankle dorsiflexion. The three scores were assigned with a point between 0 and 33, and the total score (100 points) was calculated as the sum of three scores and added by one. The definition of scores on each item was as follows: 0, no movement; 9, palpable contraction in the muscle and no movement; 14, visible movement but not in full range against gravity; 19, full range of movement against gravity but not against resistance; 25, full movement against gravity but weaker than the other side; and 33, normal power (Demeurisse et al., 1980).

#### 2.2.4 Timed Up and Go test

The Timed Up and Go (TUG) test is a commonly used test to estimate balance, gait speed, and functional ability for basic activities of daily life in the elderly (Podsiadlo and Richardson, 1991). The subjects were instructed to stand from a seated position, walk 3 m, turn back to the chair, and sit down. The duration of the whole process was recorded; a longer duration suggests a higher possibility of falls. The criterion values for detecting fall risk are presented in a range of 10–33 s (Beauchet et al., 2011).

#### 2.2.5 Range of motion

Range of motion (ROM) was assessed using goniometric measurements to quantify the initial limitations of motion, decide on appropriate interventions, and determine the effects of the interventions (Boone et al., 1978; Gajdosik and Bohannon, 1987). In this study, hip, knee, and ankle joints were evaluated using goniometers. All motions were performed passively by physical therapists, and if the values were out of the normal range, the criteria were recorded. To prevent confusion with terms such as kinematic variables and ROM, ROM in the clinical evaluation was referred to as PROM (passive range of motion) based on the measurement method. The definitions of PROM were as follows: hip, maximum or minimum angle between the trunk and thigh; knee, maximum or minimum angle between the thigh and femur; and ankle, maximum or minimum angle between the femur and foot.

### 2.3 Gait and statistical analysis

An 8-camera 3D motion capture system (Miqus Hybrid, Qualisys, Sweden) sampled at 100 Hz (Fotiadou et al., 2019; Bigoni et al., 2021) was used to collect the gait data. Three 0.5 m × 0.6-m force plates (9260AA6, Kistler, Switzerland) on the walking path were used to collect the force data while walking at 1,000 Hz. The calibration of the 3D motion capture system using the wand length was applied for 30 s before the experiment for each participant to prevent external influence. Furthermore, the standard deviation value of the calibration was set to 0.2 mm for obtaining accurate marker tracking, although the suggested value from the motion capture system is 0.5 mm. Before the start of the experiment, the participants were instructed to wear shorts for marker tracking. The participants were fitted with a Helen Hayes marker set (Lerner et al., 2014), and static trial imaging was performed. Researchers who had undergone anatomical education or physical therapists were given conservative education to ensure reliability and validity in attaching markers. Static trial imaging was carried out to capture anatomical posture, general standing posture, and feet together when in standing posture. The participants were allowed to practice walking (3–4 times) at a comfortable pace to familiarize themselves with the experimental settings. Force plate locations were adjusted so that the participants could step on them naturally during walking. In the main trials, the participants walked twice along a 10 m × 4.5-m walking path (the first time for obtaining results and the second for the preliminary trial) at a comfortable speed. Additionally, the participants were allowed to rest for 3–5 min between trials to prevent fatigue. For some marker data lost during acquisition, QTM (QTM 2023.2, Qualisys, Sweden) was used to automatically edit them using the two closest marker data available. Visual3D (C-Motion, Inc., Boyds, MD, United States) was used to preprocess the low filter at 6 Hz (Schreven et al., 2015), analyze inverse dynamics, and obtain kinetic (moments) and kinematic data on the lower limbs. Joint power was calculated using Visual3D (C-Motion, Inc., Boyds, MD, United States) as the “scalar product” of the joint moment (M) and angular velocity (w). Power can be a negative or positive quantity. When M and w are the same at a particular joint, power is a positive quantity, and energy is generated by concentric action in muscles crossing that joint. When M and w are in opposite directions, power is a negative quantity, and energy is absorbed in eccentric muscle action and/or elongation of other soft tissue crossing the joint. The data were normalized to the body weight.

All calculations were performed using MATLAB (MATLAB R2022a; MathWorks Inc., Natick, MA, United States). The maximum and minimum values of the angles were calculated for the ROM. The peak values were calculated in moments at power in the ankle, knee, and hip. We compared the data from healthy individuals and unaffected and affected sides of patients with FAC 4/5 stroke.

Statistical analyses were performed using SPSS (SPSS23; IBM Corp., Armonk, NY, United States). All datasets were tested for normality, and the nonparametric Wilcoxon rank-sum test, Wilcoxon single-rank test, and Kruskal–Wallis test were used if even one group failed to satisfy the condition of normality (Fotiadou et al., 2019; Ferraris et al., 2021).

If all groups satisfied the condition of normality, the primary endpoint, i.e., comparing biomechanical gait indices between healthy individuals and patients with FAC 4/5 stroke, was analyzed using one-way analysis of variance (ANOVA) with Bonferroni’s *post hoc* corrections. Independent *t*-tests were performed between the two groups to compare the conventional evaluation outcomes for patients with stroke depending on the FAC score.

## 3 Results

### 3.1 Conventional evaluation

**FIGURE 1:**
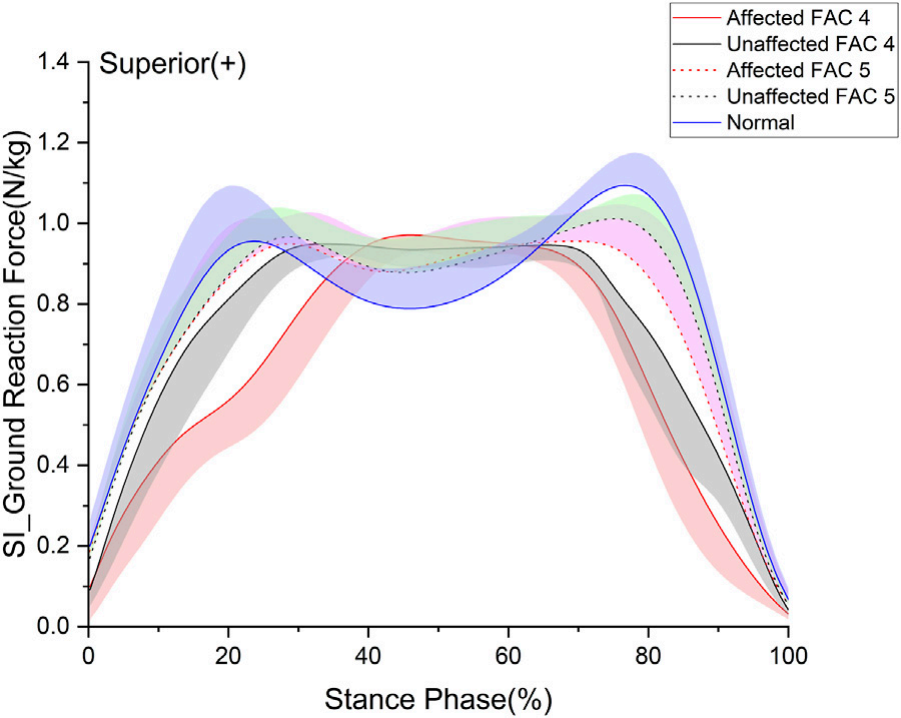
Superior–inferior ground reaction force in the stance phase. The y- and x-axes are the value normalized for body weight and the stance phase expressed as a percentile, respectively. The red and black solid lines and shaded areas indicate the patient group with FAC 4 stroke, and the red and black dotted lines and purple and chartreuse-shaded areas indicate the patient group with FAC 5 stroke. The blue solid line and shaded area indicate healthy participants. Each line represents the mean value for each group, with the standard deviation shaded and either positive or negative for better readability.

Conventional evaluation is shown in Table 2. For gait speed and stride length, the FAC 4 group was slower and shorter than normal (*p* < .001), but the FAC 5 group did not differ from normal (*p* = .102). The MAS score was 47.55 ± 1.51, close to perfect (48) for the FAC 5 group but lower in the FAC 4 group at 37.25 ± 7.98 (*p* < 0.001). The TUG score was 25.33 ± 12.34 s for the FAC 4 group, which was longer than the normal maximum of 20 s, and 11.01 ± 2.91 s for the FAC 5 group, which was in the normal range. The MI scores were 100 for the unaffected side in both FAC 4 and FAC 5 groups, and the MI scores for the affected side were 64.75 ± 10.90 and 87.55 ± 13.75 for each group, respectively. The PROMs were all fine (Figure 1).

### 3.2 Ground reaction force

Ground reaction force (GRF) is an important factor of the lower extremities measured as the loading force of the lower extremities (Zadpoor and Nikooyan, 2011). We analyzed the superior–inferior (SI) (Figure 1) and anterior–posterior (AP) components of the GRF in the sagittal plane (Table 3). In this study, a *p*-value under 0.05 is expressed as significant. The GRF in the SI direction exhibited no differences between the FACs and was significantly smaller than that of the healthy participants on the affected and unaffected sides (*p* < 0.05). The GRF in the AP direction was larger for patients of the FAC 5 group than for those of the FAC 4 group on the affected and unaffected sides. Affected sides were smaller in patients with stroke than in healthy participants (*p* < 0.001).

**TABLE 3:** Comparison of joint angle and ground reaction force feature variables.

Index	Joint/direction		FAC 4	vs.	FAC 5	FAC 4 vs.	FAC 5 vs.	Normal
Angle range of motion (ROM) (deg)	Ankle	Affected	17.0 ± 5.96	.056	20.4 ± 3.19	.024*	.342	24.1 ± 6.49
		Unaffected	20.8 ± 6.83	1.00	22.6 ± 2.62	.645	1.00	
	Knee	Affected	21.5 ± 9.47	.025*	31.0 ± 8.15	.000***	.587	34.9 ± 3.61
		Unaffected	34.4 ± 7.93	1.00	35.0 ± 4.30	1.00	1.00	
	Hip	Affected	17.0 ± 7.55	.000***	34.6 ± 8.52	.000***	1.00	37.1 ± 6.22
		Unaffected	29.6 ± 3.73	.006**	38.1 ± 5.35	.014*	1.00	
Ground reaction force MAX (N/kg)	SI	Affected	1.00 ± 0.04	1.00	1.02 ± 0.06	.012*	.012*	1.10 ± 0.07
		Unaffected	0.97 ± 0.04	.056	1.04 ± 0.04	.000***	.018*	
	AP	Affected	0.03 ± 0.02	.002**	0.09 ± 0.04	.000***	.000***	0.16 ± 0.03
		Unaffected	0.05 ± 0.02	.009**	0.11 ± 0.03	.000***	.102	

Values are presented as the mean ± SD. *p < 0.05, **p < 0.01, and ***p < 0.001.

### 3.3 Joint angle

**FIGURE 2:**
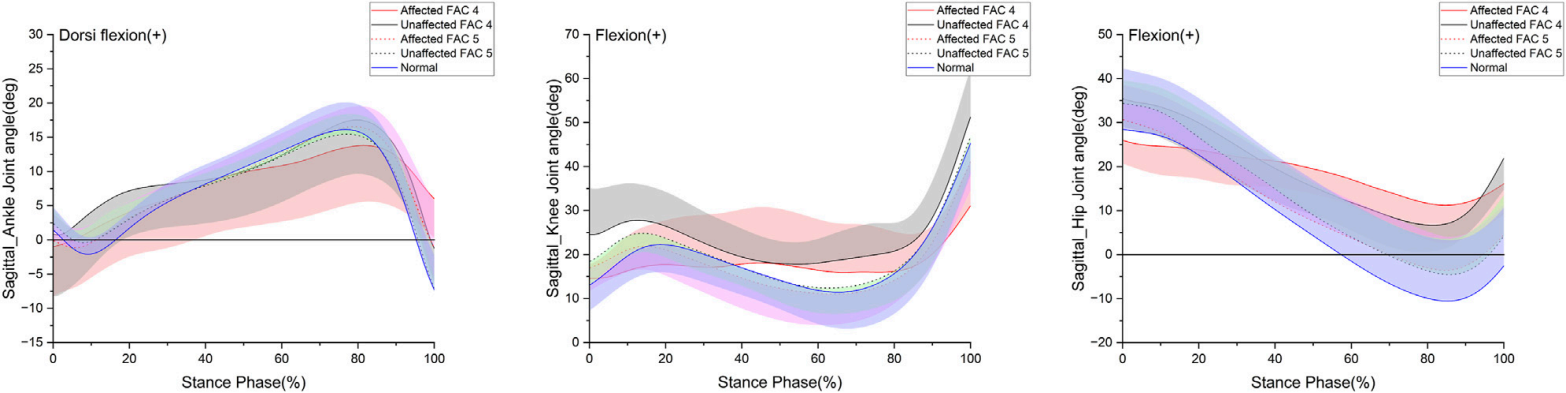
Sagittal plane–lower limb joint angle in the stance phase. The y- and x-axes are the value joint angle (degree) and the stance phase expressed as a percentile, respectively. The red and black solid lines and shaded areas indicate the patient group with FAC 4 stroke, and the red and black dotted lines and purple and chartreuse-shaded areas indicate the patient group with FAC 5 stroke. The blue solid line and shaded area indicate healthy participants. Each line represents the mean value for each group, with the standard deviation shaded and either positive or negative for better readability.

In this study, the joint angle was defined as the relative degree of lower extremities on the sagittal plane that appeared while walking. Figure 2 indicates the patterns of the sagittal plane angle in the stance phase for the major lower limb joints (ankle, knee, and hip), and Table 3 lists the statistical results. On the affected side in the FAC 4 group, the ROM for all three joints was smaller than that of the healthy participants (*p* < 0.05), whereas the ROM was only smaller in the hip joint on the unaffected side (*p* < 0.05). The FAC 5 group exhibited a significantly larger ROM than the FAC 4 group in the knee and hip joints on the affected side (*p* < 0.05) and only in the hip on the unaffected side (*p* < 0.05). In the FAC 5 group, ROM differences with the healthy group on either the affected or unaffected side were absent.

### 3.4 Joint moment

**FIGURE 3:**
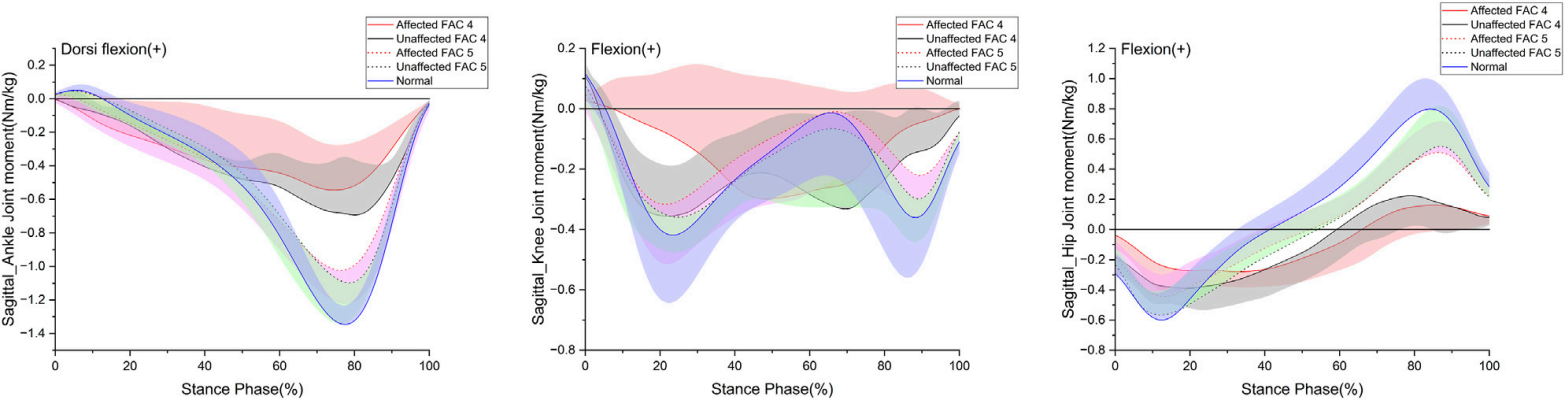
Sagittal plane–lower limb joint moment in the stance phase. The y- and x-axes are the value normalized for body weight and the stance phase expressed as a percentile, respectively. The red and black solid lines and shaded areas indicate the patient group with FAC 4 stroke, and the red and black dotted lines and purple and chartreuse-shaded areas indicate the patient group with FAC 5 stroke. The blue solid line and shaded area indicate healthy participants. Each line represents the mean value for each group, with the standard deviation shaded and either positive or negative for better readability.

Figure 3 indicates the patterns of the sagittal plane moment in the stance phase for the major lower limb joints (ankle, knee, and hip), and Table 4 lists the statistical results.

**TABLE 4:** Comparison of kinetic feature variables.

Index	Joint/direction		FAC 4	vs.	FAC 5	FAC 4 vs.	FAC 5 vs.	Normal
Dorsiflexion and flexion moment peak (Nm/kg)	Ankle	Affected	0.01 ± 0.03	.482	0.02 ± 0.03	.054	.785	0.05 ± 0.03
		Unaffected	0.03 ± 0.05	1.00	0.03 ± 0.06	.971	.200	
	Hip	Affected	0.31 ± 0.18	.055	0.54 ± 0.20	.000***	.006**	0.80 ± 0.19
		Unaffected	0.36 ± 0.19	.100	0.58 ± 0.23	.000***	.052	
Plantar and extension moment peak (Nm/kg)	Ankle	Affected	−0.61 ± 0.25	.000***	−1.05 ± 0.18	.000***	.003**	−1.34 ± 0.14
		Unaffected	−0.78 ± 0.23	.000***	−1.12 ± 0.19	.000***	.007**	
	Knee	Affected	0.35 ± 0.22	1.00	0.35 ± 0.17	.702	.609	0.48 ± 0.24
		Unaffected	0.35 ± 0.25	.741	0.46 ± 0.11	.530	1.00	
	Hip	Affected	−0.34 ± 0.13	.568	−0.46 ± .013	.004**	.076	−0.65 ± 0.25
		Unaffected	−0.31 ± 0.16	.004**	−0.58 ± 0.16	.001***	1.00	
Generation (+) power MAX (Nm/s*kg)	Ankle	Affected	0.28 ± 0.33	.032*	1.14 ± 0.51	.000***	.004**	2.14 ± 0.90
		Unaffected	0.82 ± 0.49	.083	1.55 ± 0.42	.000***	.133	
	Knee	Affected	0.20 ± 0.09	1.00	0.29 ± 0.19	.240	1.00	0.36 ± 0.21
		Unaffected	0.32 ± 0.18	1.00	0.35 ± 0.15	1.00	1.00	
	Hip	Affected	0.22 ± 0.17	.000***	0.69 ± 0.24	.000***	.036*	0.95 ± 0.23
		Unaffected	0.37 ± 0.14	.000***	0.78 ± 0.23	.000***	.251	
Absorption (−) power MIN (Nm/s*kg)	Ankle	Affected	−0.15 ± 0.08	.430	−0.32 ± 0.14	.000***	.005**	−0.66 ± 0.34
		Unaffected	−0.13 ± 0.11	.325	−0.33 ± 0.21	.000***	.015*	
	Knee	Affected	−0.27 ± 0.18	.019*	−0.89 ± 0.51	.000***	.039*	−1.39 ± 0.50
		Unaffected	−0.62 ± 0.44	.126	−1.12 ± 0.53	.007**	.609	
	Hip	Affected	−0.29 ± 0.18	.025*	−0.59 ± 0.14	.000***	.207	−0.58 ± 0.28
		Unaffected	−0.39 ± 0.24	.619	−0.54 ± 0.20	.009**	.130	

Values are presented as the mean ± SD. *p < 0.05, **p < 0.01, and ***p < 0.001.

The dorsiflexion peak at the ankle exhibited no differences with the healthy group on either the affected or unaffected side. The plantarflexion peak was significantly smaller in both FAC groups than that in the healthy group on the affected and unaffected sides (*p* < 0.01) and larger in the FAC 5 group than in the FAC 4 group (*p* < 0.001).

The knee extension moment peak showed no differences with the healthy group and in the FAC 4 and 5 groups on either the affected or unaffected side.

The hip flexion moment peak on the affected side in the FAC 4 and 5 groups was significantly smaller than that in the healthy group and in the FAC 4 group on the unaffected side (*p* < 0.05).

The hip extension moment peak was smaller in the FAC 4 group than in the healthy group on both sides (*p* < 0.01), whereas the results observed in the FAC 5 group were not different from those of the healthy group.

### 3.5 Joint power

**FIGURE 4:**
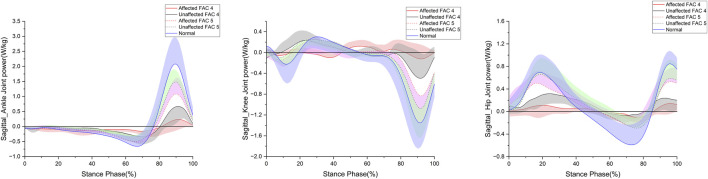
Sagittal plane–lower limb joint power in the stance phase. The y- and x-axes are the value normalized for body weight and the stance phase expressed as a percentile, respectively. The red and black solid lines and shaded areas indicate the patient group with FAC 4 stroke, and the red and black dotted lines and purple and chartreuse-shaded areas indicate the patient group with FAC 5 stroke. The blue solid line and shaded area indicate healthy participants. Each line represents the mean value for each group, with the standard deviation shaded and either positive or negative for better readability.

Figure 4 shows the patterns of the sagittal plane power in the stance phase for the major lower limb joints (ankle, knee, and hip), and Table 4 lists the statistical results.

The ankle generation power maximum was smaller in the FAC 4 and 5 groups on the affected side than in the healthy group (*p* < 0.001), larger in the FAC 5 group than in the FAC 4 group (*p* < 0.05), and smaller in only the FAC 4 group on the unaffected side than in the healthy group (*p* < 0.01). Absorption power minimum was smaller in the FAC 4 and 5 groups on the affected and (*p* < 0.001) unaffected (*p* < 0.01) sides than in the healthy group.

No differences in either the FAC 4 or FAC 5 group were observed in the knee generation power maximum. The absorption power minimum was smaller in the FAC 4 and 5 groups on the affected side than in the healthy group (*p* < 0.05), larger in the FAC 5 group than in the FAC 4 group, and smaller only in the FAC 4 group on the unaffected side than in the healthy group (*p* < 0.01).

The hip generation power maximum on the affected side was smaller in the FAC 4 and 5 groups than in the healthy group (*p* < 0.001), larger in the FAC 5 group than in the FAC 4 group (*p* < 0.001), smaller only in the FAC 4 group on the unaffected side than in the healthy group (*p* < 0.001), and larger in the FAC 5 group than in the FAC 4 group (*p* < 0.001). The absorption power minimum on the affected side was smaller only in the FAC 4 group than in the healthy group (*p* < 0.001) and was larger in the FAC 5 group than in the FAC 4 group (*p* < 0.05). On the unaffected side, the absorption power minimum was smaller only in the FAC 4 group than in the healthy group (*p* < 0.01).

## 4 Discussion

### 4.1 Conventional data

The assessments of the FAC 5 group showed no difference from healthy individuals (Table 2) in stride length or walking speed. Indeed, their PROM and MAS scores were close to perfect. The TUG score was normal at < 20 s; hence, the FAC 5 group was normal, while the MI showed a perfect score on the unaffected side and was a few points short of a perfect score on the affected side. Considering only conventional assessments, the gait recovery appears complete. However, in the case of MI, which evaluates muscle strength, hip-only flexion, knee extension, and ankle dorsiflexion were evaluated, while the remaining evaluations were mixed with various movements; therefore, there were limitations to understanding the walking behavior.

### 4.2 Kinematic data

In the FAC 5 group, kinematic parameters did not differ from those of normal individuals, suggesting that recovery was complete when considered in conjunction with conventional endpoints. Gait characteristics of patients who are hemiplegic and capable of independent gait have been previously reported. A decreased extension angle in the late stance phase and decreased flexion angle at initial contact have been reported at the hip (Moseley et al., 1993; Balaban and Tok, 2014; Sheffler and Chae, 2015). Decreased hyperextension and flexion throughout the stance phase have been reported at the knee (Moseley et al., 1993; Balaban and Tok, 2014; Sheffler and Chae, 2015), and decreased dorsiflexion at initial contact and decreased plantarflexion in the late stance phase have been reported at the ankle (Moseley et al., 1993; Sheffler and Chae, 2015). These kinematic characteristics of hemiplegic gait were consistent with our findings of the FAC 4 group; however, no difference in ROM with the healthy group was observed in the FAC 5 group; therefore, these characteristics were not observed. All kinematic gait indices of a patient in the FAC 5 group (a conventional evaluation) can be expected to be rehabilitated. In our study, all lower limb joints on the affected side exhibited a tendency for increased ROM in the FAC 5 group compared to the FAC 4 group (*p* < 0.1). This indicates that kinematic indices show differences based on the extent of functional recovery even for patients with stroke capable of independent gait. As such, when studying gait in patients with stroke, instead of the criteria such as “capable of independent gait” used in previous studies (Olney and Richards, 1996; Balaban and Tok, 2014; Wonsetler and Bowden, 2017; Shin et al., 2020; Karunakaran et al., 2022), classifying participants into further specific groups using conventional evaluations would be crucial.

### 4.3 Kinetic data

The kinetic data showed that the flexion moment and generation power of the proximal hip joint were lower than normal, and so were those of the ankle joint. Moment and power data can help understand muscle strength and movement patterns at major joints in patients with stroke and can additionally provide insights into the causes of gait disorders (Moseley et al., 1993; Olney and Richards, 1996; Balaban and Tok, 2014; Sheffler and Chae, 2015). Decreased movement of the hip joint and decreased generation power of the ankle and hip joints were previously reported as characteristics of hemiplegic gait (Moseley et al., 1993; Olney and Richards, 1996; Balaban and Tok, 2014; Sheffler and Chae, 2015). Additionally, analysis of the hip joint moment is useful for evaluating hip joint characteristics that are not apparent from kinematic data analysis alone.

The FAC 4 group showed decreased hip flexion/extension moment peaks and generation power at the ankle and hip joints. A decrease in plantarflexion moment peaks at the ankle was also observed, which might have contributed to the decreased generation power at the ankle. Previously unreported decreases in absorption forces at the ankle, knee, and hip joints were observed. Reduced absorption power was also observed in both the affected and unaffected sides. The decreased absorption power is thought to be attributed to the abnormal pattern in the affected side, although normal function was possible in the unaffected side (Moseley et al., 1993; Olney and Richards, 1996; Balaban and Tok, 2014; Sheffler and Chae, 2015).

In the FAC 5 group, a significant decrease in the generation power was observed on the affected side, although no significant decrease was observed in the hip extension moment peak, which affected the generation power. Furthermore, no decrease was observed in the generation power on the unaffected side. This suggests that power indices are better than moment indices at indicating abnormal gait characteristics in patients with stroke. The FAC 5 group was judged to have recovered normal gait function based on conventional evaluation and kinematic indices; however, the above results suggest that left–right symmetry and gait quality had not yet fully recovered. Additionally, we confirmed that even in the last stage of functional recovery, the ankle joint still lacked generation and absorption power, whereas the knee and hip joints lacked absorption and generation power, respectively. This necessitates further rehabilitation of the soleus and gastrocnemius muscles (Sheffler and Chae, 2015) involved in this power.

The absorption power of a joint is the reduction in energy by absorbing an external impact or braking the acceleration coming from the ipsilateral leg, while the generation power is the increase in energy that allows the torso to move forward (Zajac et al., 2002; McGibbon, 2003; Levinger et al., 2016; Pickle et al., 2016; Jeon et al., 2021). Therefore, the decrease in lower limb joint absorption power in the FAC 4 group indicates that the lower limb was not able to absorb sufficient shock. The FAC 5 group is also expected to have poorer shock absorption in the lower limb, with no increase in absorption at another lower joint compared to the decrease in absorption at the ankle joint. Energy that is not sufficiently absorbed by the lower limb could have been dampened in the upper limb, or it could have been used to move forward without braking. For the former, if not sufficiently dampened in the upper extremities, it can reduce head stability and increase the risk of falling (Jeon et al., 2017). The ankle and hip joints in the FAC 5 group generated less power than in the normal group, and the knee joint generation power was the same, but there was no difference in the walking speed between the normal group and the FAC 5 group. This suggests that energy, which was not sufficiently absorbed, may have been used.

We confirmed that although the main conventional assessment and gait speed (Gittler and Davis, 2018) were sufficiently normal, there was a lack of recovery of kinetic indicators, such as moment and power, which are the primary aims when organizing rehabilitation strategies for patients with stroke in clinical practice. In particular, existing classical assessments also perform MI tests to measure kinetic indicators; however, only dorsiflexion is measured in the case of the ankle, while few tests are conducted while performing movements similar to actual gait. The data also suggest that even if the recovery is complete, and the gait appears normal, there remains a lack of recovery in a complete sense; therefore, a test such as 3D gait analysis that measures actual gait remains warranted.

In addition, while ankle dorsiflexion exercises to prevent foot drop, one of the primary goals of gait rehabilitation for patients, are important (Gittler and Davis, 2018), ankle plantarflexion and hip extensor exercises that generate energy to move the body should be considered until the last stage of rehabilitation.

### 4.4 Limitations

In this study, we only compared male patients. Since gait is generally known to show sex differences (Jeon et al., 2017), further research on female participants and using stairs or slopes, where differences in biomechanical indices of gait become further prominent, must be conducted (Jeon et al., 2020). Among conventional evaluations, we only used the FAC evaluation to split and compare groups. Including other comparison groups using different conventional evaluations, such as the BBS, which is known to be markedly correlated with functional recovery from stroke (Bigoni et al., 2021), could produce further multifaceted results. Furthermore, we intend to increase the size of the patient cohort in future studies to validate our findings and follow up from the subacute phase to identify factors that affect gait recovery in the chronic phase.

## 5 Conclusion

In this study, we confirmed that although the ROM (kinematic) and conventional evaluation indices of lower extremity joints were restored to normal in the FAC 5 group, kinetic indices such as moment and power were not. We demonstrated that groups classified based on conventional evaluation exhibited several functional differences in gait even among patients capable of independent gait after post-stroke rehabilitation. Even if patients are judged to be normal in conventional evaluation and show recovery in the kinematic gait indices, they may not have complete recovery in the kinetic indices. This finding is an extension of that of previous research, wherein the patients did not show true gait recovery, including factors such as gait symmetry, although gait function appeared to have recovered in conventional evaluation (Shin et al., 2020). Thus, evaluating kinetic indices is necessary in addition to evaluating kinematic indices, and joint power may be an especially useful index. In particular, in the FAC 5 group, even in patients who were thought to have completed functional recovery of gait on classical assessment, kinetic data showed abnormalities in the hip extensor and ankle plantarflexion, which affected the generation power of the hip and ankle joints. This suggests that the recovery of function in stroke patients does not always coincide with a return to full clinical normality. Therefore, even if patients demonstrate functional gait recovery, it should not be considered the same as a healthy individual; rather, it is necessary to consider that falling at any moment remains a viable risk, and continuous patient education and rehabilitation exercises are, therefore, necessary.

## Data Availability

The raw data supporting the conclusion of this article will be made available by the authors, without undue reservation.

## Abbreviations

FAC, functional ambulation category; TUG, Timed Up and Go; MAS, motor assessment scale; MI, Motricity Index; ROM, range of motion; FIM, functional independence measure; 10MWT, 10-meter walk test; GRF, ground reaction force.
